# Increased levels of soluble triggering receptor expressed on myeloid cells sTREM1 in ICU patients with cardiovascular disease and associated organ dysfunction

**DOI:** 10.1186/cc9700

**Published:** 2011-03-11

**Authors:** S Dewan, A Varma, M Talegaonkar

**Affiliations:** 1 Fortis Escorts Heart Institute, New Delhi, India

## Introduction

sTREM1, a new receptor of the immunoglobulin superfamily, is expressed on neutrophils and monocytes/macrophages. It has been reported to be a useful marker in infectious inflammatory conditions such as sepsis, pneumonia and pancreatitis. Cardiovascular disease with shock and associated organ dysfunction in the form of acute kidney injury (AKI) and acute liver damage (LD) is a unique subset of disease conditions mediated by the inflammatory process and there may be a role of sTREM1 levels in assessing the severity of disease and prognostication of the patient. We hypothesized that the sTREM1 level may be increased in patients with cardiovascular disease and organ dysfunction and it can be used as a prognostic marker.

## Methods

A retrospective analysis of sTREM1 levels of 139 (99 males, 40 females) (*P *< 0.004) patients admitted between October 2009 and January 2010 to the ICU of our hospital. Patients with cardiovascular disease and organ dysfunction like AKI and LD were analysed. sTREM1 level >25 pg/ml was taken as abnormal.

## Results

A total of 139 patients were analysed. sTREM1 was abnormal in 82 (59%) of the patients (mean ± SD 63.26 ± 54.58) and normal in 57 (41%) patients (15.35 ± 6.10), which is highly significant (*P *< 0.0001) and correlates well with total leucocyte counts, which are (mean ± SD) 15,283 ± 6,126 for patients with abnormal sTREM1 and 13, 001 ± 6,518 for normal patients (*P *< 0.05). Out of 75 patients with coronary artery disease (CAD), 50 (61%) patients had abnormal sTREM1 levels as compared with 25 (43.9%) with normal levels (*P *< 0.046). Out of 18 patients with AKI, 15 (83.3%) had abnormal sTREM1 levels and three (16.6%) had normal levels (*P *< 0.020). Out of 15 patients with LD, 13 (84.1%) had abnormal value and two (15.9%) had normal levels (*P *< 0.017). Although patients with abnormal sTREM1 had higher mortality it was not statistically significant due to the small number of patients.

## Conclusions

sTREM1 levels rise significantly in all kinds of cardiovascular disease and associated organ dysfunction like AKI and LD. Abnormal levels are also related to higher mortality, although not statistically significantly. The level of sTREM1 can be used as a prognostic marker for patients with this kind of disease scenario. These results confirm the usefulness of sTREM1 as a biological marker for diagnosing the severity of disease.
